# Lateral flow immunoassay strips based on europium(III) chelate microparticle for the rapid and sensitive detection of *Trichinella spirali*s infection in whole blood samples of pigs

**DOI:** 10.3389/fcimb.2022.955974

**Published:** 2022-08-09

**Authors:** Xinyu Wang, Aizhe Li, Ruizhe Wang, Tianji Hou, Huixin Chen, Jing Wang, Mingyuan Liu, Chen Li, Jing Ding

**Affiliations:** ^1^ Key Laboratory for Zoonoses Research, Ministry of Education, Institute of Zoonoses, College of Veterinary Medicine, OIE Collaborating Center on Foodborne Parasites in Asian-Pacific Region, Jilin University, Changchun, China; ^2^ Changchun Institute of Biological Products Co., Ltd., Changchun, China

**Keywords:** *Trichinella spiralis*, excretory–secretory, europium(III) chelate microparticle, immunochromatographic strip, on-site detecting

## Abstract

Trichinellosis is a major food-borne parasitosis caused by ingesting raw or semi-raw meat products from pigs infected with *Trichinella spiralis* (*T. spiralis*). Although China is the largest consumer of pork in the world, the current diagnostic method of *T. spiralis* is exclusively performed in a laboratory setting, due to its complexity and laborious procedure. Here, in order to solve the detection problems in the pig breeding industry, a rapid, sensitive, and on-site serological diagnosis method was developed. The novel lateral flow immunoassay strip (ICS) is based on europium(III) chelate microparticle (ECM) to detect *T. spiralis*-specific IgG antibody in the serum and whole blood samples from pigs. The structure of the blood-filtering pad and the conjugate pad was added to the ICS, allowing for whole blood samples to be detected and enabling on-site deployment. By comparing the detection results of the serum samples and the whole blood samples, the detection limit of this method was evaluated. Thereafter, this method was used to investigate *Trichinella* infection in Chongqing, Sichuan, Inner Mongolia, Guangxi, and Liaoning provinces of China, and the results were almost consistent with the standard method of artificial digestion. Taking advantage of its user-friendly procedure, short detection time (3 min), and sensitivity, the ECM-ICS could be employed for monitoring the epidemic of *Trichinella* infection and ensuring meat safety.

## Introduction

Trichinellosis is a neglected food-borne zoonotic parasitic disease caused by nematodes of the genus *Trichinella*, which infects more than 150 species of animals, including humans (Dupouy-Camet, 2000; Pozio, 2007; Murrell, 2016). According to the International Commission on Trichinellosis (ICT), a total of 65,813 people in the world were diagnosed with trichinellosis as a result of eating raw or semi-raw meat from 1986 to 2009 (Murrell and Pozio, 2011). Furthermore, in 2015, nearly 40,000 cases of *Trichinella* infections were reported in China (Yang et al., 2020). In recent years, outbreaks of trichinellosis have occurred several times in remote areas of central and western China where people have a habit of eating uncooked pork (Bai et al., 2017). Hence, there is an urgent need to carry out epidemiological monitoring of trichinellosis and to strengthen the prevention and control of *Trichinella* infections.

According to the recommendation of the ICT and the World Organization for Animal Health (OIE), serological methods are an acceptable method of detecting *Trichinella* infection in animals (Gamble et al., 2004; Bruschi et al., 2019). The most widely used serological method is enzyme-linked immunosorbent assay (ELISA) based on excretory–secretory (ES) antigens (Gamble et al., 1988; Kapel and Gamble, 2000; Gomez-Morales et al., 2009). Depending on their accuracy and sensitivity, ELISAs serve as a reliable method to detect *Trichinella* in a diagnostic lab. However, ELISA-based detection is plagued by lengthy, laborious, and user-unfriendly procedures.

The immunoassay strip (ICS) is a sensitive, convenient, and rapid option for detecting pathogen infection. More recently, ICS has played a key role in the field of detecting severe acute respiratory syndrome coronavirus 2 (SARS-CoV-2) infections, further promoting the advantage of ICS in epidemic disease outbreaks (Chen et al., 2021; Zhang et al., 2021). Traditional ICS methods employ the use of gold nanoparticles, quantum dots, or fluorescent microspheres (Liang et al., 2017; Willen et al., 2018; Lee et al., 2019). These different ICS methods have previously been developed for the diagnosis of *Trichinella* infection, albeit using serum samples from pigs and in a laboratory setting (Zhang et al., 2006; Fu et al., 2013; Xu et al., 2021). Europium(III) chelate microparticle (ECM) is a fluorescent microsphere-based ICS and has the advantages of sensitivity and specificity. Additionally, ECM benefits from long fluorescence bursts and is less susceptible to stray light and long Stokes displacement ICS (Hu et al., 2017). No previous literature has been published on investigating the potential of ECM-based ICS for on-site detection of *Trichinella* infection and its promise as a powerful tool for epidemic monitoring.

In our previous work, we developed an ECM-based ICS capable of detecting *T. spiralis*-specific IgG antibody in pig serums; the sensitivity, specificity, and cross-reaction were evaluated (Wang et al., 2021). Aiming for clinical whole blood samples, we simplified the procedure and improved the effectiveness of the ECM-based ICS. A blood-filtering pad and a conjugate pad were incorporated into the ICS, which retained high sensitivity and accuracy. More importantly, the novel ICS can be used for whole blood samples detected and is potentially suitable for a wider range of applications.

## Materials and methods

### Reagents and instruments

Bovine serum albumin (BSA) and Tween-20 were purchased from Solarbio (Beijing, China). 1-Ethyl-3-(3-dimethylaminopropyl)carbodiimide hydrochloride (EDC) and N-hydroxysulfosuccinimide (NHS) were purchased from Tokyo Chemical Industry (TCI Shanghai, China). COOH-modified europium nanoparticles (EuNPs) were purchased from Thermo Fisher (USA). Mouse anti-pig monoclonal IgG antibodies and rabbit anti-goat IgG antibodies were purchased from Beijing Biolab Technology (Beijing, China). Goat anti-rabbit IgG was purchased from Cell Signal Technology (USA). The NC membrane (CN140) was purchased from Sartorius (USA), and Ultra-15 3 kDa, Millipore 135, and Millipore 90 centrifugal filters were purchased from Millipore (USA). The sample pads; the conjugate pads SB06, SB08, VL78, VL98, RB45, and RB65; the blood filter membranes; the absorbent pads; and the plastic backing were obtained from Jinbiao Biotech (Shanghai, China). The BSA protein assay kit was obtained from Beyotime Biotechnology (Shanghai, China).

The XYZ3060 dispenser was obtained from BioDot (USA). The TRF fluorescence quantitative analyzer was obtained from Weice Biotech (Nanjing, China). A UV lamp was obtained from Shenzhen Feike Technology (Shenzhen, China).

### Preparation of ES antigens

The preparation of muscle larvae (ML) ES antigens follows the procedures described previously (Bai et al., 2016; Wang et al., 2020). A total of eight specific pathogen-free (SPF) SD rats were orally infected with 4,500 *T. spiralis* (T1 ISS534) ML each. Subsequently, the rats were euthanized at 35 dpi and muscle samples were used to recover *T. spiralis* ML by following the artificial digestion method. Then, the ML was washed several times with 0.9% saline solution until clean and resuspended with serum-free RPMI-1640 medium including antibiotics (100 U/ml of penicillin and 100 μg/ml of streptomycin); the culture condition was 37°C for 18 h in 5% CO_2_. Finally, the culture supernatant was concentrated by Ultra-15 3 kDa centrifugal filters, and the concentration of the protein was measured by BCA kits.

The preparation of preadult worm (PAW) ES antigens was performed according to the procedures described in Sun et al. (Sun et al., 2015). An additional eight SPF SD rats were infected with 10,000 *T. spiralis* ML each and euthanized at 6 h post-infection. The tissue of the small intestine was harvested from the abdominal cavity and was put in 0.9% saline solution (containing 200 U/ml of penicillin and 200 μg/ml of streptomycin) at 37°C for 2 h after being split longitudinally. The cultivation and preparation of PAW-ES antigens were performed the same as the ML-ES antigens.

### Pig and serum samples

Four Landrace pigs aged 10 weeks were inoculated with 400, 600, 600, and 800 doses of *T. spiralis* ML, and a total of 60 serum samples were collected at 0, 7, 9, 11,13, 15, 17, 19, 21, 25, 30, 35, 45, 60, and 90 dpi. Five Landrace pigs aged 10 weeks were inoculated with 0, 200, 600, 1,000, and 10,000 *T. spiralis* ML, and a total of 20 serum samples and 20 whole blood samples were collected at 0, 21, 25, 30, and 35 dpi. The whole blood samples collected from pigs infected with 10,000 *T. spiralis* at 60 dpi were served as a standard positive control.

A total of 1,500 whole blood samples were collected from slaughterhouses in Chongqing, Sichuan, Inner Mongolia, Guangxi, and Liaoning provinces of China.

### Preparation of the fluorescent probe

The preparation of the fluorescent probe was followed by the previous protocol of Yan et al. (Yan et al., 2021). Firstly, 20 μl of ECM was centrifuged at 18,000×g for 10 min, and subsequently, the liquid supernatant was discarded. Then, 20 µl of 1 mg/ml EDC, 20 µl of 1 mg/ml NHS, and 200 µl of MES (50 mmol/L, pH 6.0) were mixed with the ECM and incubated for 1 h. Then, the mixture was centrifuged at 18,000×g for 10 min to remove the phosphate and glycerol. Once removed, 20 μg of PAW-ES antigens and ML-ES antigens at the ratio of 1:1 in 200 µl of PBS buffer (50 mmol/L, pH 8.0) were added to it. The mixture was incubated for 3 h, followed by a centrifuging step at 18,000×g for 10 min; the supernatant was discarded. Then, the mixture was blocked by 200 µl of 5% BSA overnight at 4°C. Finally, the ECM-ES fluorescent probes were dissolved in a preserved buffer (100 mmol/L of PBS containing 1.5% BSA and 1.5% ProClin). The method of ECM conjugated with goat anti-rabbit IgG was the same as the ECM-ES.

### Preparation of the immunochromatographic strips

The ICS consisted of an absorbent pad, nitrocellulose membrane (NC membrane), conjugate pad, blood filter pad, and baseboard. Afterward, the ECM-ES with ECM-goat anti-rabbit IgG was mixed at 3:1. The fluorescent probes were sprayed on a conjugate pad using the XYZ3060 dispenser. Meanwhile, mouse anti-pig monoclonal IgG (1 mg/ml) and rabbit anti-goat IgG (1 mg/ml) were sprayed on the position of the testing line (T-line) and the control line (C-line) of the NC membrane by the XYZ3060 dispenser, respectively. Following a 2-h drying step at 37°C, the five parts were assembled forming the ICS. Finally, the ICS was cut at a width of 3.8 mm and placed in a white cassette.

### Optimization of ICS

The addition of a conjugate pad is a key step toward the commercialization of the novel ICS. In order to achieve a perfect release of fluorescent particles by the ICS, the relevant reaction steps of the conjugate pad were optimized. Firstly, the materials of the conjugate pads SB06, SB08, VL78, VL98, RB45, and RB65 made of glass fiber and polyester fiber were assessed for suitability for use in the novel ICS. Secondly, the fluorescent particles conjugated with ES antigens at a ratio of 10:1, 20:1, 30:1, and 40:1 were evaluated on the novel ICS. Thirdly, the fluorescent particles sprayed on the conjugate pad at the amounts of 0.4, 0.8, and 1.2 µg were evaluated on the ICS. In order to achieve desirable incubation times, multiple NC membranes, CN140, Millipore 135, and Millipore 90 were tested on the novel ICS. The serum obtained from the pigs infected with 10,000 *T. spiralis* ML at 60 dpi was used to optimize the reaction conditions in this part. For all conditions, visual results were observed by a 365-nm UV lamp 3 min after the samples were added to the ICS.

### Determination of the dilution limit of the whole blood samples by ICS

After collecting the whole blood samples using anticoagulation tubes, these samples were detected by ICS at the dilution of 1:50, 1:100, 1:200, 1:400, 1:800, 1:1,600, 1:3,200, 1:6,400, 1:12,800, and 1:25,600. The whole blood samples from pigs infected with 10,000 *T. spiralis* ML at 60 dpi and uninfected pigs were chosen as the standard positive and negative controls. Visual results were observed by a 365-nm UV lamp, and the fluorescent signal was analyzed by a fluorescent reader 3 min after the samples were added.

### Sensitivity evaluated in ICS employing the conjugate pad

To evaluate the sensitivity of ICS after employing the conjugate pad, the serum samples from pigs infected with 400, 600, 600, and 800 *T. spiralis* ML at 0–90 dpi were detected by ICS. In these three infectious doses, the pork samples contain approximately 0.5–1.5 larvae per gram (LPG). Previous studies indicated that the detection limit of the gold standard method (artificial digestion) is 1–3 LPG (Gamble, 1998). If the detection effect is better than the digestion method, it can be assumed that the sensitivity of the method is higher than that of the current gold standard.

### Comparison of whole blood and serum samples detected by ICS

To effectively detect the whole blood samples, a blood filter pad was incorporated into the ICS, which could filter out the red blood cells. To evaluate the limit of detection of the ICS for whole blood samples and serum samples, two kinds of samples collected from pigs infected with 0, 200, 600, 1,000, and 10,000 *T. spiralis* ML at 0, 21, 25, 30, and 35 dpi were analyzed by ICS. Following a 3-min incubation period, visual results were observed by a 365-nm UV lamp, and the fluorescent signal was analyzed by a fluorescent reader.

### Investigation of *Trichinella* infection in China by ICS

The ultimate aim of this research was to apply this method to the clinical detection of *Trichinella*. A total of 1,500 whole blood samples were collected from slaughterhouses in the villages of Chongqing, Sichuan, Inner Mongolia, Guangxi, and Liaoning provinces in China where *Trichinella* infection has been widely reported. The novel ICS detection method’s accuracy and its potential clinical application were verified using artificial digestion.

### Statistical analysis

The fluorescence intensity of the T-line of ICS was analyzed by a fluorescent reader, and the results were expressed as the mean ± SD using GraphPad Prism8.

## Results

### The principle of the ICS

**Figure 1:**
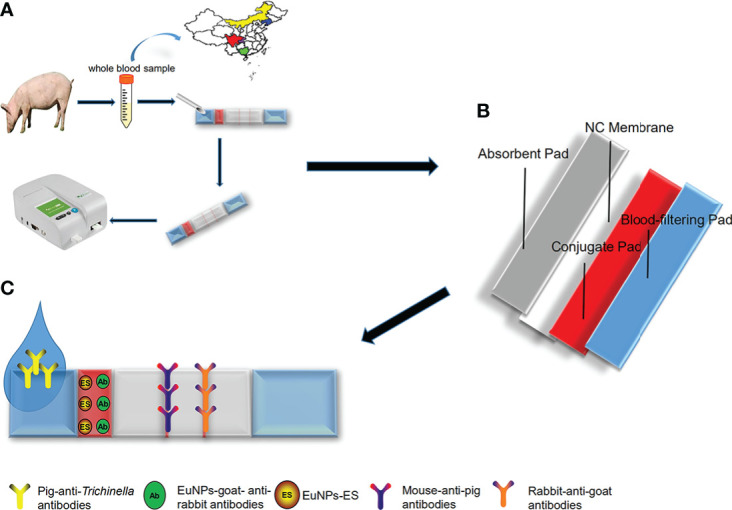
The schematic of europium(III) chelate microparticle-immunoassay strip (ECM-ICS). **(A)** Detecting the whole blood sample by ECM-ICS. **(B)** Schematic depiction of the construction of the ECM-ICS. **(C)** Immunochromatographic process of the ECM-ICS.

The principle of the ICS can be considered a classical indirect detection method; the ECM-ES fluorescent probes served as capture probes and the ECM-goat anti-rabbit IgG fluorescent probes served as indicative probes. Firstly, *T. spiralis*-specific IgG antibodies were captured by fluorescent ECM-ES probes, and these, in turn, react with mouse anti-pig IgG antibodies on the T-line. Meanwhile, the fluorescent ECM-goat anti-rabbit IgG probes were immobilized on the C-line by rabbit anti-goat IgG antibodies. Therefore, when ICS detected a positive sample, both the T-line and the C-line had a fluorescent signal. When ICS detected a negative sample, only the C-line had a fluorescent signal. Due to the addition of a blood filter membrane into the ICS, both serum samples and whole blood samples could be detected (**Figure 1**).

### Optimization of the ICS

**Figure 2:**
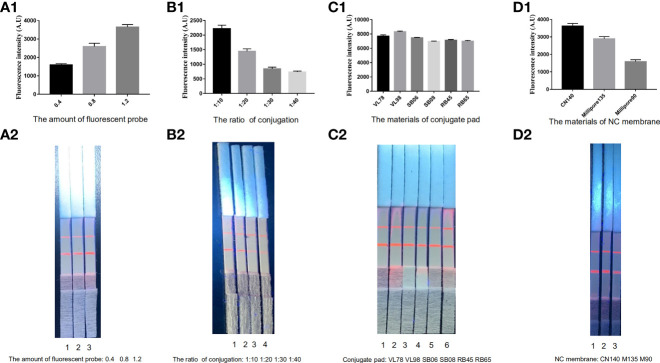
Optimization of the ECM-ICS. **(A)** Fluorescent particles sprayed on conjugate pads 1–3: 0.4, 0.8, and 1.2 µg. **(B)** The rate of ES antigens conjugated with fluorescent particles 1–4: 1:10, 1:20, 1:30, and 1:40. **(C)** The materials of conjugate pads 1–6: VL78, VL98, SB06, SB08, RB45, and RB65. **(D)** The materials of NC membranes 1–3: CN140, Millipore 135, and Millipore 90. ECM-ICS was analyzed by the TRF reader **(A1, B1, C1, D1)**. Visual results of the ICS under ultraviolet light **(A2, B2, C2, D2)**.

Optimizing the reaction conditions of the conjugate and NC membrane pads in the ICS conformed to the principle that fluorescent particles were realized adequately and the T-line broke out the stronger fluorescent signal. The use of the 0.8-µg fluorescent particles at the ratio of 10:1 conjugated with ES antigens, which was sprayed on the conjugate pad, yielded the best results. Moreover, it was determined that the VL78 conjugate pad and the CN140 NC produced the desired result (**Figure 2**).

### The dilution limit of the whole blood samples detected by ICS

**Figure 3:**
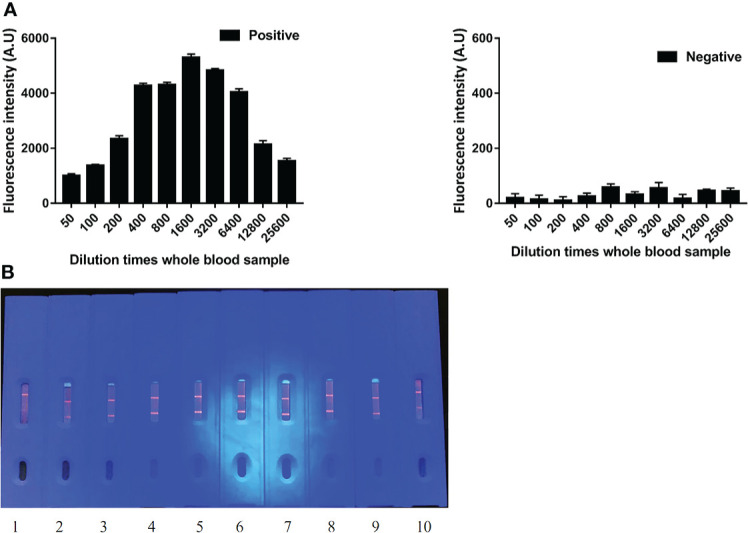
The dilution limit of whole blood samples by ECM-ICS. **(A)** ECM-ICS was analyzed by the TRF reader. **(B)** Visual results of ICS under ultraviolet light. 1, 2, 3, 4, 5, 6, 7, 8, 9, and 10 represent the whole blood samples diluted 50, 100, 200, 400, 800, 1,600, 3,200, 6,400, 12,800, and 25,600 times.

The fluorescence intensity of ICS was the strongest when whole blood samples were diluted 1,600 times. Moreover, the visual ranking was consistent with the fluorescence analysis results. The novel ICS was able to produce a fluorescence signal for positive samples, even when diluted 25,600 times. Considering the fact that whole blood samples cannot be diluted too much in practical applications, we recommend diluting the sample 1,600 times (**Figure 3**).

### Sensitivity evaluated in the ICS employing the conjugate pad

**Figure 4:**
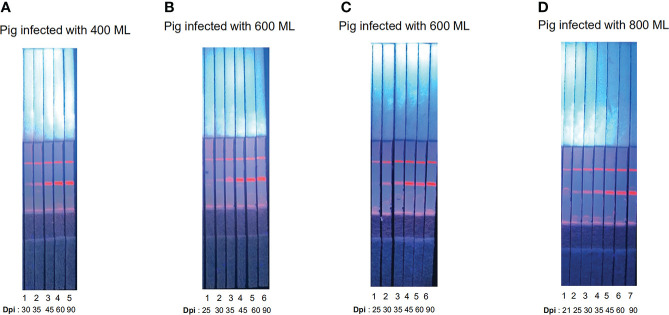
Serum samples detected by ECM-ICS. **(A)** Samples from pigs infected with 400 *Trichinella*. **(B)** Samples from pigs infected with 600 *Trichinella*. **(C)** Samples from pigs infected with 600 *Trichinella*. **(D)** Samples from pigs infected with 800 *Trichinella*. **(A1–5)** 30, 35, 45, 60, and 90 dpi; **(B1–6)** 25, 30, 35, 45, 60, and 90 dpi; **(C1–6)** 25, 30, 35, 45, 60, and 90 dpi; **(D1–7)** 21, 25, 30, 35, 45, 60, and 90 dpi.

The fluorescent particles were fully released and the T-line produced a strong fluorescent signal; in the pigs infected with 400, 600, 600, and 800 ML, seroconversion was first detectable by ICS at 30, 25, 25, and 21 dpi, respectively (**Figure 4**). Compared with other results (Gondek et al., 2018), the novel ICS could detect early infection even the pigs were infected with low dose of Trichinella, which indicated ICS has high sensitivity. Furthermore, the fluorescence intensity of the T-line increased with days post-infection (**Figure 4**).

### Comparison of the ICS for detecting whole blood and serum samples

**Figure 5:**
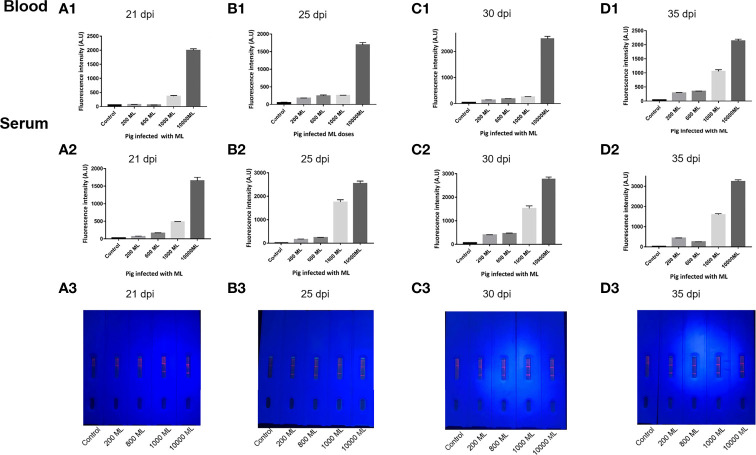
Comparison of ECM-ICS for detecting whole blood and serum samples. **(A)** Samples from pigs infected with *Trichinella* at 21 dpi. **(B)** Samples from pigs infected with *Trichinella* at 25 dpi. **(C)** Samples from pigs infected with *Trichinella* at 30 dpi. **(D)** Samples from pigs infected with *Trichinella* at 35 dpi. **(A1, B1, C1, D1)** ECM-ICS for detecting whole blood samples analyzed by the TRF reader; **(A2, B2, C2, D2)** ECM-ICS for detecting serum samples analyzed by the TRF reader;**(A3, B3, C3, D3)** the whole blood samples detected by ECM-ICS and the visual results under ultraviolet light.

As shown in **Figure 3C**, in pigs infected with 200, 600, 1,000, and 10,000 ML, seroconversion was first detected by ICS at 25, 25, 21, and 21 dpi, respectively. Although the signal of whole blood is slightly weaker than that of serum, the results of both samples were consistent when visually rated. More interesting, regardless of the sample type, the fluorescence intensity of the T-line increased with the dose of infection, which indicated that the level of antibody produced by the host is positively correlated with the dose of infection (**Figure 5**).

### Investigation of *Trichinella* infection in China by ICS

The performance of the ICS for detecting whole blood was shown, and the process of detection could be completed within 3 min without specialized equipment or the need for specially trained personnel (**Video 1**). Based on this method, five suspected trichinosis endemic areas were screened using the novel test strip. Results showed that five whole blood samples were detected as positive by ICS and three pork samples were detected as positive by artificial digestion (**Table 1**).

**Table 1:** The results of investigating *Trichinella* infection.

Method	Positive	Negative	Total
Artificial digestion	3	1,497	1,500
ECM-ICS	5	1,495	1,500

## Discussion

Developing an on-site detection method for *Trichinella* infections is of critical importance, which will contribute to epidemiological investigations and monitoring of potential epidemic outbreaks. Based on our previous research, this study is a further research for the exploration of ICS based on ECM detection in clinical application. Here, we demonstrated that the seroconversion time detected by means of the developed novel ICS for use with whole blood samples was consistent with serum samples. Moreover, we used this method to complete an epidemiological survey of five provinces in China.

Optimization of the conjugate pad material employed for the novel ICS played an important role in allowing the use of the ICS to be more convenient and user-friendly. Firstly, our result shows that attention should be given to the application of fluorescent particles to the conjugate pad. The spraying of fluorescent particles required exact precision because excessive fluorescent particles will not react with the antibody and cause high fluorescence background on the NC membrane. In contrast, insufficient fluorescent particles will result in an insufficient reaction between the antigen and the antibody. Furthermore, the selection of the NC membrane is another key factor in developing the ICS method. If the detection reaction rate is defined as R, it is reflected by the affinity of the antibody for the antigen K and the concentration of the antigen (Ag) and the antibody (Ab) on the NC membrane (see equation). Different NC membranes have different pore sizes, which determine the crawling speed of the antibody on the membrane, thereby affecting the reaction time of the antigen-antibody. Therefore, on the premise of ensuring the specificity of the ICS, increasing the incubation time of the antigen with the antibody on the NC membrane could improve the sensitivity of the ICS.


R=K[Ag][Ab]

The use of the blood-filtering pad makes ICS drastically decrease the test time, which allows the ICS to detect the whole blood samples. Despite the addition of this extra structure, the limit of detection of the novel ICS was not affected. Moreover, compared with the previous study (Gondek et al., 2018), the novel ICS could detect early anti-*Trichinella* antibodies in pigs, and this may have been due to the method employing the use of the PAW-ES antigens. We were able to confirm previous findings, which reported that the use of early antigens could shorten the diagnostic window of *Trichinella* infections (Liu et al., 2016; Wang et al., 2017a; Wang et al., 2017b). Due to the ML-ES being easily acquired and widely used in the detection of *Trichinella* infection, we determined to combine the two antigens at a ratio of 1:1 in this coupling system.

Investigating *Trichinella* infection in China is of great significance. The difference in the results between the ICS and artificial digestion may be due to the host interacting with the worms, causing a response that results in the expulsion of the parasites in low doses, or due to the habit of feeding insecticides in rural areas. In summary, this study provides a useful tool for performing on-site detection of *Trichinella* infection, having the advantages of being sensitive, rapid, and convenient. The use of a conjugate pad and a blood filter membrane facilitated the use of the novel ICS in clinical applications. Our novel detection method is superior compared to more mainstream serological detection methods for the detection of early *Trichinella* infection.

## Data availability statement

The original contributions presented in the study are included in the article/**Supplementary Material**. Further inquiries can be directed to the corresponding authors.

## Ethics statement

This study was reviewed and approved by Administration of Affairs Concerning Experimental Animals in China, The protocol was approved by the Institutional Animal Care and Use Committee of Jilin University (20170318).

## Author contributions

XW, AL, RW, and JD participated in the data collection and drafted the manuscript. CL, ML, and JW participated in designing this study and revising the manuscript. TH, HC, and XW participated in collecting the samples. All authors contributed to the article and approved the submitted version.

## Funding

This study was supported by the National Natural Science Foundation of China (NSFC31872467, 31902290) and the Program for JLU Science and Technology Innovative Research Team (2017TD-32).

## Conflict of interest

Author ML was employed by Changchun Institute of Biological Products Co., Ltd.

The remaining authors declare that the research was conducted in the absence of any commercial or financial relationships that could be construed as a potential conflict of interest.

## Publisher’s note

All claims expressed in this article are solely those of the authors and do not necessarily represent those of their affiliated organizations, or those of the publisher, the editors and the reviewers. Any product that may be evaluated in this article, or claim that may be made by its manufacturer, is not guaranteed or endorsed by the publisher.
